# Delayed effects of the relative humidity on the outpatient visits of dry eye disease in Shanghai, China: effect modification by green and blue space

**DOI:** 10.7189/jogh.15.04142

**Published:** 2025-05-05

**Authors:** Yiming Xu, Liwei Zhang, Yun Yang, Wushuang Wang, Han Zhao

**Affiliations:** 1 The Second Xiangya Hospital of Central South University, Department of Ophthalmology, Changsha, China; 2 Hunan Clinical Research Center of Ophthalmic Disease, Changsha, China; 3 Eye & ENT Hospital, Fudan University, Department of Ophthalmology, Shanghai, China; 4 Chinese Academy of Medical Sciences, NHC Key laboratory of Myopia and Related Eye Diseases, Shanghai, China; 5 Shanghai Key Laboratory of Visual Impairment and Restoration, Shanghai, China

## Abstract

**Background:**

Few studies have examined the influence of relative humidity (RH) on dry eye disease (DED), particularly in the context of the urban environment. The objective of this study was to investigate the influence of RH on the frequency of outpatient visits for DED among patients residing in diverse urban settings.

**Methods:**

Data pertaining to outpatient visits, together with data on the DED, meteorological factors and air pollutants in Shanghai for the period 2019–2023, were retrieved. To analyse the nonlinear connection and lag impact between RH and outpatient visits for DED patients, a distributed lag nonlinear model (DLNM) was fitted.

**Results:**

A total of 159 832 cases were utilised for the investigation. The results of the single-day lag pattern demonstrated a significant correlation between elevated RH exposure and DED. Lower RH was a substantial risk factor for DED on the basis of the cumulative-day effect pattern. Male were more susceptible to DED, and patients aged 0–18 years presented more stable performance in response to RH influences. In the cumulative-day lag pattern, the green space groups, the RR of the >60 years subgroup was greater than that of the other subgroups, and in the blue space groups, the RR of the 19–60 subgroup of blue space ^low^ was higher than that of the blue space ^high^.

**Conclusions:**

Reduced RH increases the relative risk of DED outpatient visits and suggests that a specific disease burden is associated with low RH exposure. Additionally, green and blue spaces in urban areas influence RH, which in turn affects the outpatient visits of DED at different ages.

Dry eye disease (DED) is a common ocular surface disease and is a general term for a wide range of disorders characterised by abnormal tear quality, quantity or kinetics resulting in decreased tear film stability, accompanied by ocular discomfort and/or damage to ocular surface tissues from any cause [1]. Symptoms associated with this condition include redness, dryness and itchiness of the eyes, grittiness, foreign body sensation, eye fatigue, and reduced visual acuity [2,3]. Dry eye disease affects millions of people worldwide, with an estimated global prevalence ranging from 5 to 50%, with varying degrees of severity depending on the population [4,5]. This condition can have a significant effect on the physical and mental health of patients, reducing their overall quality of life [6]. Furthermore, DED can also affect productivity in the workplace by making prolonged computer use or reading more difficult, reducing tolerance to certain environments and decreasing working hours [7,8].

Dry eye disease has a broad spectrum of potential causes [6]. It frequently results from environmental triggers or pharmaceutical agents (including over-the-counter medications such as antihistamines) [9]. In a Spanish statistical study, a 1033-patient model was used to demonstrate the impact of local climate change on the development of DED [9]. Environmental factors, such as wind, low relative humidity (RH), high altitude, high temperature, and air pollution, strongly influence DED. Among these factors, RH plays a pivotal role in DED [10]. In addition to individual risk factors, environmental factors such as RH and temperature have been identified as potential contributors to the prevalence, signs and symptoms of DED [11,12]. Adequate tear production and stability are essential for maintaining a healthy tear film [13]. A Korean study with data from 33 cases revealed that there is a significant positive correlation between RH and temperature and that it affects changes in DED indicators in patients [13]. In particular, low RH has been demonstrated to impair tear film stability and has been repeatedly linked to a higher outpatient visits amount of DED [14,15].

The urban environment has a close impact on people's lives today. The green space and blue space in a city play various roles [16]. Blue space is an area dominated by surface water bodies, whereas green space usually refers to areas with vegetation, such as parks and urban forests [17]. The green space and blue space in cities play important roles in temperature adjustment, air pollutant absorption and RH control [18]. In recent years, China's industrialisation and urbanisation have continued to grow, and people's living environments have changed dramatically. Industrialisation has changed the shape of cities and the distribution of green space and blue space, which in turn affects the overall urban climate. Changes in air quality are significant among these changes. The eye surface is directly exposed to the air, and changes in the different components of the air significantly affect the health of the eye surface [19]. A study in Shenyang, a traditional industrial city in northeastern China, showed that in a model of 10 809 patients, changes in air quality caused by industrial development directly affected corneal discomfort and the outpatient visits of DED. Similarly, in a study of 25 818 subjects in Taiwan, transportation emissions due to industrialisation increased the outpatient visits of DED [15]. Reduced green and blue space in both cities has led to a rise in the number of patients with DED as a result of a reduction in the regulatory capacity of local environments, such as transpiration from green spaces, and water vapor evaporation from waters.

Green space has many positive effects, including improving human comfort by lowering temperatures and increasing RH [20]. In addition, green space has the potential to mitigate and regulate air pollution [21]. Blue space has long been seen as a linking strategy for the urban heat island effect, with its own local climate regulation capabilities [22]. Both can effectively regulate RH, and studying the distribution of both may provide insight into the factors that contribute to the pathogenesis of DED within cities. It can be hypothesised, based on the results of previously available studies [21,22], that urban green and blue spaces may mitigate DED risks by regulating local RH through evapotranspiration and evaporative cooling. However, real-world evidence on their combined effects remains scarce, particularly in rapidly urbanising regions like Shanghai.

RH can be defined as the ratio of the quantity of water vapor present in the air to the maximum quantity of water vapor that the air can contain at a given temperature. The RH is typically expressed as a percentage, with a higher percentage indicating a more humid air-water mixture. A reduction in RH has been demonstrated to result in accelerated tear evaporation, referred to as tear film break-up time (TBUT), and augmented ocular surface staining in controlled experimental settings [23–25]. Furthermore, the utilisation of moisture-resistant goggles has been documented to increase DED symptoms and diminish tear evaporation rates [26]. Nevertheless, the impact of RH on specific DED parameters has not been comprehensively investigated in authentic, real-world scenarios and beyond the confines of laboratory environments [27]. At present, there is a paucity of data pertaining to the impact of RH on DED in China. Clinicians commonly underestimate the impact of environmental factors, as the treatment of DED is largely focused on conventional artificial tears and other therapeutic approaches [27].

In recent years, urban development in Shanghai has led to the fragmentation of green spaces, with limited public accessibility to the original green areas [28]. Simultaneously, the expansion of urban construction has further reduced blue spaces, weakening the capacity of water bodies to regulate the urban environment [29]. In this study, we obtained a substantial amount of data from outpatient clinics for DED patients at the Eye & ENT Hospital of Fudan University in Shanghai, China. The nonlinear relationship between RH and the outpatient visits of DED was assessed distributed lag nonlinear modelling (DLNM). Furthermore, the influence of different subgroups (sex, age and season) on this relationship was also analysed.

## METHODS

### Study region

This retrospective time-series study analysed daily DED outpatient visits from 2019 to 2023, linking them to meteorological and environmental data. The city of Shanghai is located in eastern China, at the mouth of the Yangtze River. The territory is bounded by the Yangtze River in the north, the East China Sea in the east, Hangzhou Bay in the south, and Jiangsu Province and Zhejiang Province in the west. Shanghai is located in the alluvial plain of the Yangtze River Delta; the terrain is open and low, with a subtropical monsoon climate, and the largest river is the Huangpu River. The total area of Shanghai is 6340.5 km^2^. As of the end of 2022, the population of Shanghai was estimated to be 24.75 million. Shanghai's subtropical monsoon climate and high level of urbanisation make it a particularly relevant case for studying the interaction between RH and DED. The city's climate is characterised by significant seasonal variations in humidity, while rapid urbanisation has led to environmental changes that may exacerbate DED symptoms. These unique conditions provide an ideal setting to explore the impact of RH on DED in a real-world, highly urbanised context.

### Data collection

The study did not involve patient privacy; therefore, no ethical approval was needed. In addition, the study was conducted at the Eye & ENT Hospital of Fudan University. Information on patients with DED was extracted from the hospital information system.

All participants were diagnosed by experienced ophthalmologists, who conducted comprehensive examinations and evaluated the presence of symptoms. The DED diagnosis was based on the Chinese Dry Eye Expert Consensus (2020). Additionally, data pertaining to individual characteristics were collected, including sex (female, male), age (0–18 years, 19–60 years, >60 years), address, date of visit, and whether the visit was a first or subsequent visit. Only return visits of more than 30 days were included in the final analysis. The day-by-day RH (%), from 1 January 2019 to 31 December 2023, was obtained from the Shanghai Municipal Environmental Protection Bureau through fixed meteorological monitoring stations.

### Regional indicators

**Figure 1:**
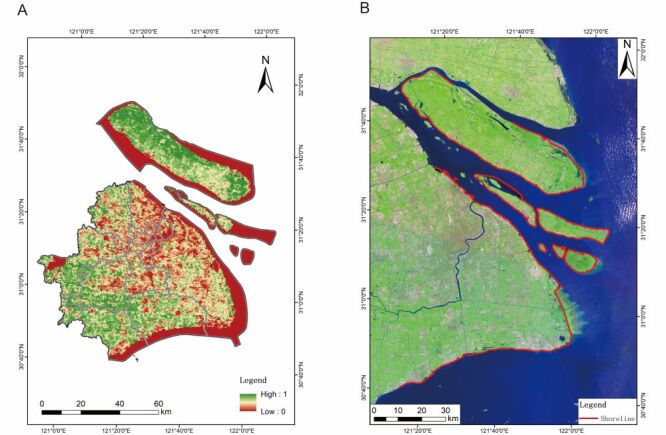
Distribution of green space (**Panel A**) and blue space (**Panel B**) in Shanghai.

The normalised difference vegetation index (NDVI) is an indicator of vegetation cover calculated via satellite remote sensing data and is particularly suitable for monitoring and assessing changes in vegetation. The NDVI is calculated by comparing reflectance values in the near-infrared band with those in the infrared band [18]. The Chinese NDVI maximum data set was calculated via the Google Earth Engine remote sensing cloud computing platform via Landsat 5/8 remote sensing images from the US Landsat satellites, and we obtained the data from Shanghai from this data set. From this, we estimate the area in green space (**Figure 1**, Panel A). The proportion of open water bodies in a residential area or county is representative of blue space exposure [30]. On the basis of the geographic information system (GIS) platform, we used the distance of the arithmetic centre position of each borough from the ocean as the value for calculation (**Figure 1**, Panel B), which was used as an indicator of patient blue space exposure when confirming patient exposure [31]. NDVI quantifies vegetation density, directly linked to evapotranspiration and RH modulation. Distance to water bodies serves as a proxy for blue space accessibility, influencing local humidity levels.

### Statistical analysis

The mean, standard deviation, minimum, 25th percentile, 50th percentile, 75th percentile, and maximum are used to express daily outpatient visits and RH, respectively. The potential correlations between the variables were evaluated via Spearman's correlation coefficient.

To assess the exposure-response relationship between RH and visits to DED clinics by children and adolescents, a time series model was developed (Methodological Framework for Assessing the Impact of Relative Humidity on Dry Eye Disease Outpatient Visits in the **Online Supplementary Document**). Given that the daily visits for DED patients were Poisson distributed, a case-crossover approach based on a Poisson generalised linear model and DLNM was employed to investigate the delayed effect of RH on DED visits [32]. In particular, we constructed an exposure-response dimension fit via the natural cubic spline (NS) function and an exposure-lag dimension fit. Furthermore, the NS function was employed to construct a cross-basis function for the fitted exposure-lag-response independent variable (RH). To demonstrate the lag effect over a longer period of time, it was necessary to set an upper limit on the number of lag days. Considering that dry eye disease is a chronic condition and to explore the long-term effects of blue space and green space on dry eye through the modulation of relative humidity, we have set the lag days at 28 days [33,34].

Furthermore, the exposure-response relationship between RH and DED clinic visits of patients in different subgroups was evaluated [35]. Subgroups were analysed according to sex (female, male), age (0–18 years, 19–60 years, >60 years) and season (warm season, cold season). The warm season is defined as the period between April and September, whereas the cold season is the opposite, occurring between October and March [36]. The statistical significance of the differences between the various subgroups was assessed using the following formula [35]:



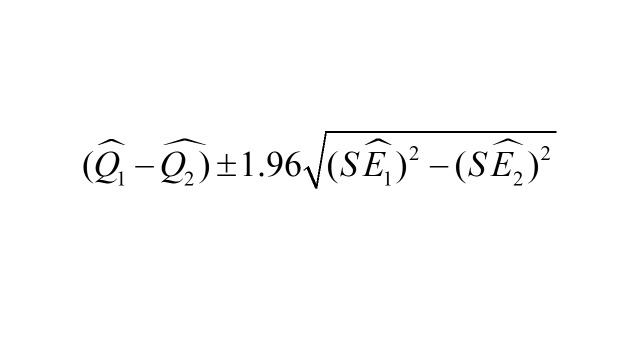



In this context, *Q*_1_ and *Q*_2_ refer to the estimates for these subgroups, while SE_1_ and SE_2_ refer to the standard errors. Furthermore, to validate the disease burden of DED patients, we estimated the attributable fraction (AF) and attributable number (AN) of DED patients with cumulative exposure to RH at different lags *day*_t_. Attributable function represents the proportion of disease cases (here, DED) that can be attributed to a specific exposure (in this study, cumulative RH exposure). It quantifies the theoretical reduction in disease burden if the exposure were eliminated or reduced. Attributable number indicates the absolute number of disease cases (DED) directly attributable to the exposure (RH). It is calculated by multiplying the AF by the total number of cases. The formulas for the AF and AN are provided below for reference [37]:

AF_t_ = 1 − exp(−β*_t_*)

AN_t_ = AF_t_ × N_t_

In this context, β_t_ represents the relative risk associated with *day_t_*, whereas *N*_t_ denotes the *day_t_* count of DED in children and adolescents.

To ascertain the robustness of the model, sensitivity studies were conducted. First, the df for RH was altered to df = 3–5. Second, the df for time was modified to df = 6–8. Third, the lag period was extended to 31 days (equivalent to one month) in accordance with the findings of a previous study [38].

All analyses were conducted via R software (version 4.2.2, Vienna, Austria) with the following packages: 'dlnm', 'splines', 'mgcv', 'tsModel', ‘nlme’, ‘reshape2’ and ‘ggplot2’. A *P*-value of less than 0.05 indicates statistical significance (two-tailed).

## RESULTS

### Descriptive analysis

**Table 1** shows the daily information for patients with DED from 1 January 2019 to 31 December 2023. Our cohort contained 159 832 cases in total, and the daily mean and standard deviation (SD) were 87.531 and 61.482, respectively. There were 46 558 outpatient visits by males for DED, almost half of those visits by females (113 274 cases). In terms of age, the highest group was 19–60 years, with 101 642 cases, followed by >60 years, with 54 627 cases, and the smallest group was 0–18 years. Moreover, 79 278 and 80 554 cases were identified during the warm season and cold season, respectively.

**Table 1:** Description summary of daily outpatient visits of patients with dry eye disease in Shanghai, 2019 – 2023

Variables	Sum	Mean	SD	Minimum	P_25_	Median	P_75_	Maximum
**Daily outpatient visits**	159 832	87.531	61.482	0	30	84	145	230
**Sex**								
Female	113 274	62.034	44.264	0	21	59	102	161
Male	46 558	25.497	17.927	0	9	24	41	76
**Age**								
0–18	3563	1.951	1.984	0	0	1	3	12
19–60	101 642	55.664	38.395	0	20	53	90	158
>61	54 627	29.916	23.774	0	7	28	49	97
**Season**								
Warm season (April to September)	79 278	86.643	59.506	0	31	90	142	230
Cold season (October to March)	80 554	88.424	63.424	0	28	81	146	222
**Residential area (Green space)**								
Green space ^high^	86 443	47.340	34.005	0	16	45	78	135
Green space ^low^	73 389	40.191	28.371	0	14	39.5	66	108
**Residential area (Blue space)**								
Blue space ^high^	70 957	38.859	27.785	0	13	37.5	64	104
Blue space ^low^	88 875	48.672	34.483	0	17	46	80	140

SD – standard deviation, P_25_ – 25th percentile, P_75_ – 75th percentile

**Table 2** presents descriptive data on RH, meteorological factors, and air pollutants. During this period, the daily mean RH ranged from 39% to 98%. The daily average mean RH, wind speed, air pressure, temperature, and precipitation were 76.689%, 3.660 m per second (m/s), 1016.074 hectopascal (hPa), 18.632°C and 3.201 millimetres (mm)/24-hour (h), respectively. The 24-hour average air pollution concentrations of particulate matter less than 2.5 µm (μm) (PM_2.5_), particulate matter less than 10 μm (PM_10_), sulphur dioxide (SO_2_), nitrogen dioxide (NO_2_), and carbon monoxide (CO) and the 8-hour average ozone (O_3_) concentration were 33.014 µg (μg)/m^3^, 45.848 μg/m^3^, 13.857 μg/m^3^, 36.005 μg/m^3^, 0.919 μg/m^3^, and 16.940 μg/m^3^, respectively.

**Table 2:** Description summary of daily air pollutant concentration, relative humidity, and other meteorological factors in Shanghai, 2019–2023

Variables	Mean	SD	Minimum	P_25_	Median	P_75_	Maximum
**Air pollutant concentration (24 h average)**							
PM_2.5_ (μg/m^3^)	33.014	23.936	2	16	25	42	155
PM_10_ (μg/m^3^)	45.848	27.239	0	28	38	58	308
SO_2_ (μg/m^3^)	13.857	22.203	1	5	6	8	119
NO_2_ (μg/m^3^)	36.005	21.820	0.500	21	32	46	119
CO (μg/m^3^)	0.919	1.293	0	0.520	0.630	0.800	11
**Air pollutant concentration (8 h average)**							
O_3_ (μg/m^3^)	76.940	34.940	0	52	74	98	274
Meteorological factors							
Mean temperature (°C)	18.632	8.621	-4.5	11	19	26	35.5
Relative humidity (%)	76.689	10.387	39	70	78	84	98
Wind speed (m/s)	3.660	1.408	1	3	3	4	14
Air pressure (hPa)	1016.074	9.177	987	1008	1017	1023	1042
Precipitation (mm/24 h)	3.201	8.998	0	0	0.070	1.890	117.860

CO – carbon monoxide, h – hour, hPa – hectopascal, m^3^ – cubic metre, mm – millimetre, μg – microgram, NO_2_ – nitrogen dioxide, O_3_ – ozone, PM_10_ – particulate matter less than 10 μm, P_25_ – 25th percentile, P_75_ – 75th percentile, PM_2.5_ – particulate matter less than 2.5 μm, SD – standard deviation, SO_2_ – sulphur dioxide

Figure S1, Panels A–B in the **Online Supplementary Document** display the daily time series distributions of meteorological factors, air pollutants, and outpatient visits for DED patients. Seasonal and periodic trends were observed in the distributions of the five meteorological parameters and the six air pollutants, as well as in the total number of daily outpatient visits for DED. Among them, the mean temperature exhibited the same pattern as the RH and precipitation, which were reduced in winter and more elevated during the summer. The pattern of changes in air pressure is the opposite. The remaining meteorological factors and air pollutants fluctuated widely.

The changes in meteorological parameters and air pollution in the warm season and cold season are depicted by violin plots and boxplots (Figure S2, Panels A–B in the **Online Supplementary Document**). The PM_2.5_, PM_10_, SO_2_, NO_2_, and CO concentrations increased in the cold season, whereas O_3_ increased in the warm season. In addition, the mean temperature, RH, wind speed and precipitation were greater in the warm season. The air pressure was higher in the cold season. Additionally, compared with that in the warm season, the number of cases of daily DED was slightly greater in the cold season (Figure S2, Panel C in the **Online Supplementary Document**).

Using Spearman’s method, we additionally evaluated the relationships between climate variables and air pollution. The highest correlation was found between air pressure and temperature. The details of the coefficients are shown in Table S1 in the **Online Supplementary Document**. We conducted sample size testing to examine the relationship between RH and DED outpatient visits. The results showed a significant correlation with a *P*-value of 0.02233 (<0.05). The correlation coefficient (cor = −0.0534666) indicates a negative relationship, suggesting that higher relative humidity may be associated with lower outpatient visits (Figure S3 in the **Online Supplementary Document**).

### Lag-response analysis between RH and DED

Figure S4, Panels A–B in the **Online Supplementary Document** present the exposure-lag-response effects of RH associated with DED outpatient visits at various lag days. Outpatient visits for DED were significantly inversely correlated with RH exposure in a nonlinear pattern.

Figure S5 in the **Online Supplementary Document** displays the association of RH with the RR of DED patients admitted at different lag days. In the single-day lag pattern, these results demonstrated a substantial correlation between higher RH exposure and DED at lag 6 (RR = 1.047; 95% CI = 1.008, 1.087) to lag 20 (RR = 1.042; 95% CI = 1.010, 1.076), with the peak RR at lag 13 (RR = 1.087; 95% CI = 1.052, 1.123) (Table S2 in the **Online Supplementary Document**). These results demonstrated that lower RH exposure was substantially responsible for DED on the basis of the cumulative-day lag effect pattern at lag 0–10 (RR = 1.538; 95% CI = 1.056, 2.238) to lag 0–28 (RR = 3.907; 95% CI = 2.040, 7.485), with the peak RR at lag 28 (Table S2 in the **Online Supplementary Document**).

### Subgroup analysis

**Figure 2:**
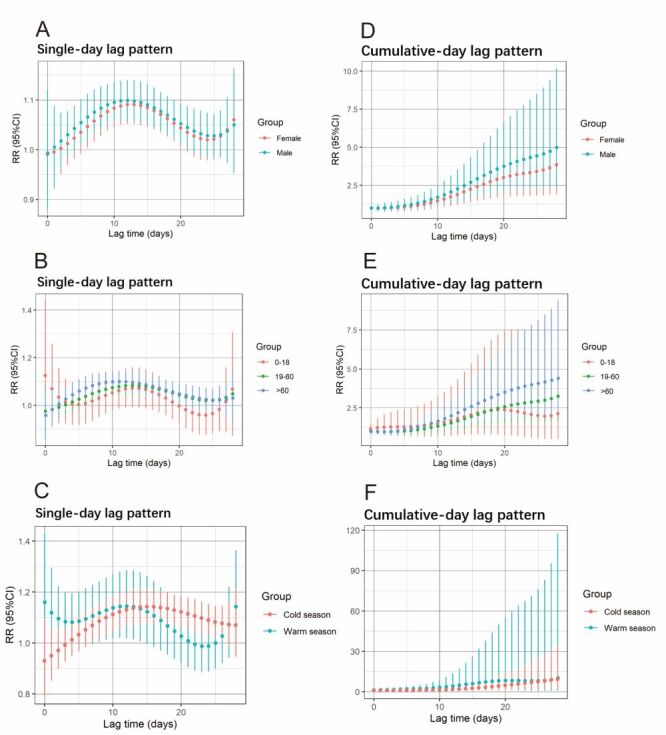
Relationships between RH and the relative risk of outpatient visits for DED, categorised by patient sex, age, and season and living place over various lag days. Single-day lag pattern (**Panel A**) and cumulative-day lag effect pattern (**Panel D**) in female and male. The single-day lag pattern (**Panel B**) and cumulative-day lag effect pattern (**Panel E**) at 0–18 years, 19–60 years, and >60 years. The single-day lag pattern (**Panel C**) and cumulative-day lag effect pattern (**Panel F**) in the warm season and cold seasons. CI – confidence interval, RH – relative humidity, RR – relative risk.

We carried out a stratified analysis by sex, age, and season that revealed significant associations between RH exposure and DED risk, particularly in males, individuals aged >60 years, and during the warm season under a single-day lag pattern (**Figure 2**, Panels A–C; Table S3–5 in the **Online Supplementary Document**). Males exhibited consistently higher DED risk (RR) than females from lag 1 (RR = 1.016; 95% CI = 0.916, 1.127) to lag 26 (peak RR = 1.027 at lag 13), while females showed a delayed significant RR at lag 28 (RR = 1.061, peak at lag 13) with lower overall risk. Age-based analysis highlighted elevated RR in the 0–18 years subgroup at lag 0–2 (peak RR = 1.077 at lag 0), though weaker than the elderly (>60 years), who demonstrated sustained risk from lag 4 (RR = 1.050) to lag 13 (peak RR = 1.101 at lag 12). The 18–60 years subgroup displayed modest associations (peak RR = 1.082 at lag 13), underscoring age-related susceptibility. Seasonal variations further differentiated risk patterns: warm season exposure correlated with an immediate peak RR = 1.146 at lag 0, declining through lag 21, whereas cold season exposure showed a delayed peak RR = 1.127 at lag 15, persisting to lag 28. These trends emphasise demographic and seasonal disparities in RH-associated DED risk, with heightened vulnerability among males, the elders, and during warm season.

For cumulative risk, similar trends were observed across subgroups, with males exhibiting higher risk (RR = 4.549; 95% CI = 2.267, 9.129 at lag 0–28) compared to females (RR = 3.674; 95% CI = 1.881, 7.175) (**Figure 2**, Panel D). The >60 years subgroup showed significant RH-DED associations at lag 0–10 (RR = 1.866; 95% CI = 1.211, 2.875) and lag 0-28 (RR = 5.201; 95% CI = 2.457, 11.007). While the 0–18 years subgroup displayed greater sensitivity than the 19–60 years group at shorter lags (lag 0–0 to 0–6, peak RR = 1.077, 1.152), the 19–60 years subgroup surpassed at longer lags (lag 0–20 to 0–28, RR = 2.742, 3.495) (**Figure 2**, Panel E). Seasonal analysis revealed comparable cumulative RR patterns between warm and cold seasons overall, but warm season RR (*e.g*. lag 0–10: RR = 3.066; lag 0–22: RR = 6.296) exceeded cold season values at specific intervals (**Figure 2**, Panel F), highlighting nuanced temporal and demographic variations in RH-DED risk.

For both the overall population and various subgroups, Table S6 displays the cumulative-day lag effects of exposure to extremely low RH (5th) and extremely high RH (95th) on hospitalisations for DED (Table S6 in the **Online Supplementary Document**). We found that in most subgroups, as time increased, the RH increased during DED. Mostly, there was a trend of increasing impacts over time. In contrast, the cumulative effect of extremely low RH on DED was weaker than that of extremely high RH on DED, but both effects were significant. At lag 0–1, the extremely low RH and extremely high RH subgroups were not obviously correlated.

### Attributable fraction and attributable number analysis

**Table 3** presents the AF and AN of RH in different subgroups of DED patients. We chose five time points (lag 0–1, lag 0–7, lag 0–14, lag 0–21, and lag 0–28) to compare the correlation coefficients. Among these five time points, the AFs of DED patients were 0.023 (95% CI = −0.22, 0.264), 0.219 (95% CI = −0.137, 0.543), 0.678 (95% CI = 0.316, 0.9), 0.888 (95% CI = 0.566, 0.989), and 0.945 (95% CI = 0.646, 0.998) at lag 0–1, lag 0–7, lag 0–14, lag 0–21 and lag 0–28, respectively. Moreover, the AN rates of DED patients were 3628 (95% CI = −35 162, 42 154), 34 951 (95% CI = −21 894, 86 810), 108 419 (95% CI = 50 547, 143 812), 141 876 (95% CI = 90 402, 158 119), and 151 100 (95% CI = 103 315, 159 588) at lag 0–1, lag 0–7, lag 0–14, lag 0–21 and lag 0–28, respectively. For the sex subgroup, males had the highest AF (0.971, 95% CI = 0.718, 1.000) at lag 0–28. Similar to these results, the >60 years subgroup also had the highest AF (0.985, 95% CI = 0.767, 1.000) at lag 0–28. In addition, the cold season subgroup had the highest AF (0.998, 95% CI = 0.717, 1), 0.998 (95% CI = 0.717, 1.000) and AN (80 431, 95% CI = 57 771, 80 554) values at lag 0–28.

**Table 3:** Attributable fractions and attributable numbers with 95% confidence interval of daily admission for DED over different lag days stratified by patient’s sex, age and season

Groups	Total case, n	Attributable fractions, % (95% CI)					Attributable numbers, n (95% CI)				
		**Lag 0-1**	**Lag 0-7**	**Lag 0-14**	**Lag 0-21**	**Lag 0-28**	**Lag 0-1**	**Lag 0-7**	**Lag 0-14**	**Lag 0-21**	**Lag 0-28**
Total	159 832	0.023 (−0.220, 0.264)	0.219 (−0.137, 0.543)	0.678 (0.316, 0.900)	0.888 (0.566, 0.989)	0.945 (0.646, 0.998)	3628 (−35 162, 42 154)	34 951 (−21 894, 86 810)	108 419 (50 547, 143 812)	141 876 (90 402, 158 119)	151 100 (103 315, 159 588)
Sex											
*Female*	113 274	0.021 (−0.229, 0.269)	0.191 (−0.176, 0.528)	0.646 (0.261, 0.889)	0.866 (0.506, 0.987)	0.931 (0.586, 0.998)	2355 (−25 945, 30 467)	21 602 (−19 912, 59 854)	73 207 (29 521, 100 649)	98 105 (57 289, 111 762)	105 460 (66 348, 113 038)
*Male*	46 558	0.027 (−0.233, 0.284)	0.286 (−0.093, 0.618)	0.750 (0.391, 0.940)	0.930 (0.642, 0.996)	0.971 (0.718, 1.000)	1239 (−10 842, 13 218)	13 322 (−4318, 28 761)	34 933 (18 216, 43 754)	43 320 (29 900, 46 390)	45 220 (33 446, 46 544)
Age (in years)											
*0–18*	3563	0.112 (−0.412, 0.599)	0.157 (−0.592, 0.790)	0.468 (−0.443, 0.959)	0.580 (−0.546, 0.994)	0.519 (−0.787, 0.998)	401 (−1468, 2133)	559 (−2111, 2817)	1667 (−1577, 3418)	2067 (−1944, 3543)	1851 (−2803, 3555)
*19-60*	101 642	0.010 (−0.240, 0.259)	0.151 (−0.216, 0.495)	0.599 (0.200, 0.864)	0.840 (0.455, 0.982)	0.918 (0.547, 0.997)	1020 (−24 396, 26 337)	15 332 (−21 926, 50 271)	60 876 (20 318, 87 806)	85 406 (46 258, 99 777)	93 257 (55 613, 101 339)
*>60*	54 627	0.039 (−0.238, 0.312)	0.350 (−0.053, 0.685)	0.822 (0.476, 0.970)	0.960 (0.709, 0.999)	0.985 (0.767, 1.000)	2128 (−13 003, 17 064)	19 102 (−2883, 37 444)	44 879 (26 005, 52 986)	52 462 (38 717, 54 577)	53 808 (41 903, 54 625)
Season											
*Warm*	79 278	0.236 (−0.164, 0.594)	0.633 (−0.032, 0.957)	0.963 (0.264, 1.000)	0.995 (0.144, 1.000)	0.998 (−0.099, 1.000)	18 746 (−13 034, 47 105)	50 217 (−2553, 75 866)	76 381 (20 968, 79 278)	78 895 (11 442, 79 278)	79 150 (−7821, 79 278)
*Cold*	80 554	−0.052 (−0.372, 0.274)	0.070 (−0.448,0.562)	0.675 (−0.003, 0.970)	0.972 (0.493, 1.000)	0.998 (0.717, 1.000)	−4155 (−29 993, 22 038)	5607 (−36 116, 45 282)	54 337 (−240, 78 166)	78 305 (39 724, 80 553)	80 431 (57 771, 80 554)

CI – confidence interval

### Synergistic effect of RH on green space and blue space

**Figure 3:**
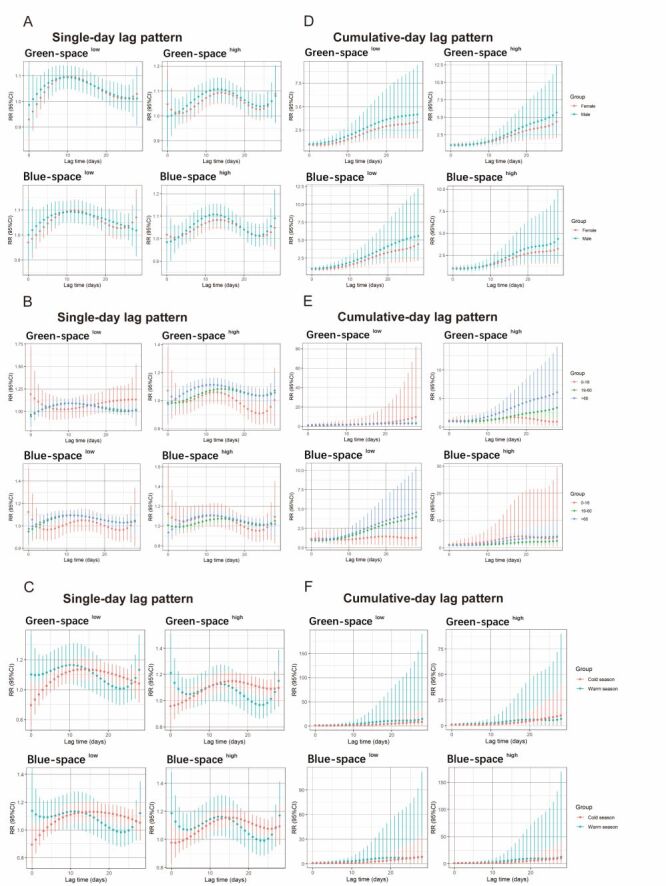
Relationships between RH and the relative risk of outpatient visits for DED, classified by patient sex, age, and season, over various lag days in the categories of low and high green space and blue space. Single-day lag pattern (**Panel A**) and cumulative-day lag effect pattern (**Panel D**) in female and male. The single-day lag pattern (**Panel B**) and cumulative-day lag effect pattern (**Panel E**) at 0–18 years, 19–60 years, and >60 years. The single-day lag pattern (**Panel C**) and cumulative-day lag effect pattern (**Panel F**) in the warm and cold season. DED – dry eye disease, RH – relative humidity.

This study is the first to examine how RH affects outpatient visits for DED and whether there are any differences in how living places affect different subgroups of patients. In this modification assessment, we divided the subgroups into two categories with two groups (green space ^high^ and green space ^low^, blue space ^high^ and blue space ^low^) on the basis of the median concentration of exposure to green space or blue space. **Figure 3** shows the RR of RH exposure to outpatient visits for DED according to sex, age and season in the blue and green space categories within a 28-day lag period. On the basis of the single-day lag pattern, we found that the relationship between RH exposure and DED was significant in the female and male subgroups in the blue space ^high^ and green space ^high^ categories. In the blue space ^high^ category, the male subgroup had the highest RR at lag 13 (**Figure 3**, Panel A), and the highest RR of the green space ^high^ subgroup occurred at lag 12. There was no significant correlation in the age subgroup (**Figure 3**, Panel B). For the seasonal subgroup, the effects of RH exposure on outpatient visits for DED have the same regularity. In both the green space group and the blue space group, the warm season subgroup was significant in the first 10-day lags, and after 15 days, the cold season subgroup became more significant (**Figure 3**, Panel C).

In the cumulative-day lag pattern, the effects of RH exposure on hospitalisations for DED were significant in the male subgroup for the two categories (**Figure 3**, Panel D). Importantly, in the green space groups, the RR of the >60 years subgroup was greater than that of the other subgroups, and in the blue space groups, the RR of the 19–60 subgroup of blue space ^low^ was higher than that of the blue space ^high^ (**Figure 3**, Panel E). There were no significant differences for the seasonal subgroups in the green space and blue space categories (**Figure 3**, Panel F).

### Sensitivity assessment

In this sensitivity assessment, we changed the parameter settings of time (df = 6–8), meteorological factors (df = 3–5), and air pollutants (df = 3–5), and our model remained stable (Figure S6–7 in the **Online Supplementary Document**). Additionally, the major outcomes under the exposure-lag pattern were not significantly altered when the maximum lag days were increased from 28 to 31 (Figure S8 in the **Online Supplementary Document**). According to the above results, the model used in this study was robust.

## DISCUSSION

The outpatient visits of DED are increasing, highlighting a growing public health concern [39]. RH refers to the ratio of the actual water vapor pressure in the air to the saturated water vapor pressure at the prevailing air temperature, which reflects how far the air is from saturated air [40]. Blue spaces (*e.g*. urban water bodies like lakes and rivers) increase RH through evaporation. Due to their high thermal capacity, they absorb heat during the day and release it slowly at night, potentially hindering environmental cooling but elevating RH [41]. For green spaces, vegetation enhances RH via transpiration, while linear green spaces (*e.g*. street trees) reduce wind speed, minimising the spread of dry air and slowing RH loss [42]. Clinical studies indicate that each 1% increase in humidity decreases DED symptom scores by 0.431 points [43]. Thus, the appropriately elevated RH in the environment has a role to play in manifesting the interference of DED.

This study is the first large-scale investigation of the impact of RH on DED outpatient visit rates. A total of 159 832 cases were used to study this topic over the period of 2019–2023. Our findings suggest that a decrease in RH increases the risk of DED after controlling for air pollutants and meteorological factors, including PM_2.5_, PM_10_, NO_2_, SO_2_, CO, O_3_, wind speed, temperature, precipitation, and barometric pressure. In addition, the effect of RH was more prominent in the sex model. Overall, our findings provide a comprehensive understanding of the effect of RH on DED hospitalisation and provide a reference for the relationship between the urban environment and the health of the population.

Stratification analysis data revealed that, most of the time, males had a greater outpatient visits of DED than females did, which may be related to work style and average eye hygiene habits, and the RR due to sex grouping in the sample was almost unaffected by green space and blue space. In the age groupings, we find that different age groups are more significantly affected by green space and blue space. In the cumulative-day ag pattern of the blue space groups, a greater distance from the sea resulted in lower RH, causing an increase in the RR in the >60 years and 19–60 years groups, whereas in different environments, the 0–18 years group was more stable. In addition, when the seasonal relationships were compared, the cold season and warm season groups were relatively more stable than the age and sex groups were, the RR values were almost unaffected by green space and blue space, and there were distinct characteristics among the seasons. The shorter period of lag days (approximately less than 10 days) shows a greater RR of the warm season on DED, and in the following ten days (approximately 15 days later), it is the cold season that causes the increased impact, which may be related to the seasonal climatic characteristics of the two seasons. By considering the values of RR with the performance of different subgroups in the subgroups of green space and blue space, we can see that moderating the inner-city environment can have an impact on the onset of DED, especially when it is grouped by age.

In developing countries, the acceleration of anthropogenic climate change, such as urbanisation and industrialisation, affects vulnerable groups, especially the elderly [44]. Therefore, climatic factors should be integrated with known clinical treatments in the management of patients with DED. In cases where it is difficult to control the overall environment, altering the environment of localised living spaces, such as maintaining appropriate RH and temperature of the surrounding air (*e.g*. in the patient's home or workplace), like the use of indoor humidifiers, ensures good eye health, especially if the RH of the patient living in these places is low [9]. More geodemographic-specific studies that use RH to track the impact of current climate change risks on DED are needed to establish strategies for controlling environmental risk factors that are appropriate for regional climatic characteristics and sociodemographic conditions. In urban construction and planning, increasing the proportion of green space and blue space is conducive to regulating the local climate and has an effect on the improvement of DED, as well as other diseases [21]. In the case of Shanghai, there are very practical implications for building wetland parks near industrial areas, enforcing minimum green space ratios in new developments, and restoring reclaimed water bodies, among others.

There are several highlights of this study. Our study is the largest available model of RH and the onset of DED, with high statistical value. We not only obtained the RR and 95% CI of RH on DED but also investigated the attributional role of RH. In addition, we considered the relationships between green space and blue space both for DED and RH, and the values for green space and blue space were selected and calculated relatively objectively to assess the environmental factors of DED onset more comprehensively. We also consider the lag period so that a relatively long lag (four weeks) is more conducive to the long-term assessment of data.

This study has several limitations. First, our study focused only on the city of Shanghai, and we collected data from the city of Shanghai. In the process of acquiring blue space, exposure data are more difficult to analyse accurately than green space data are, as China has not provided a more stable and accurate source of statistics on intraurban water bodies. Moreover, Shanghai as a whole has low vegetation cover, so the impact of green space is relatively less significant than that of blue space. We currently do not have data on the correlation between urban proxy heat island effects and residential area income. Additionally, there are limitations, such as the lack of direct measurements of indoor humidity or individual behaviours (*e.g*. air conditioning usage). We will further discuss these limitations in the discussion section and address them in future studies. On the patient side, we have less access to comprehensive information about the patients, and the basic information used in the data statistics is the basic information of the patients at the time of admission, such as the time of contact with green space or blue space, living habits, etc., which may also affect the results of the study and the correlation test of the data. There is also a need to further improve the methodology for assessment in the context of research realities, such as statistics on the number of lagging days.

## CONCLUSIONS

In summary, our findings suggest that reduced RH increases the relative risk of DED outpatient visits and suggest that a specific disease burden is associated with low RH exposure. Additionally, we found that green and blue spaces in urban areas influence RH, which in turn affects the outpatient visits of DED at different ages. Our study provides important insight into the relationship between DED pathogenesis and the environment.

## Additional material


Online Supplementary Document
