# Supplementary material for: Delayed effects of the relative humidity on the outpatient visits of dry eye disease in Shanghai, China: effect modification by green and blue space
Source: J Glob Health. 2025 May 5;15:04142. doi: 10.7189/jogh.15.04142 (PMC12050904; doi:10.7189/jogh.15.04142)

Supplement to: Y Xu, L Zhang, Y Yang, W Wang, H Zhao, Delayed effects of the relative humidity on the outpatient visits of dry eye disease in Shanghai, China: Effect modification by green and blue space. J Glob Health. 2025;15:04142.

Yiming Xu[1, 2]* (https://orcid.org/0009-0006-4108-0352), Liwei Zhang[1, 2]* (https://orcid.org/0000-0002-4349-5360), Yun Yang[3, 4, 5], Wushuang Wang[3, 4, 5], Han Zhao[1, 2] (http://orcid.org/0000-0002-5803-505X)

[1]The Second Xiangya Hospital of Central South University, Department of Ophthalmology, Changsha, China

[2]Hunan Clinical Research Center of Ophthalmic Disease, Changsha, China

[3]Eye & ENT Hospital, Fudan University, Department of Ophthalmology, Shanghai, China

[4]Chinese Academy of Medical Sciences, NHC Key laboratory of Myopia and Related Eye Diseases, Shanghai, China

[5]Shanghai Key Laboratory of Visual Impairment and Restoration, Shanghai, China

*Joint first authorship.

Correspondence to:
Name Han Surname Zhao
Institution The Second Xiangya Hospital of Central South University
Address No. 139, Renmin Middle Road, Changsha, Hunan, 410011, China.
Country China

email zhaohan_0601@csu.edu.cn

Methodological Framework for Assessing the Impact of Relative Humidity on Dry Eye Disease Outpatient Visits

To ensure the accuracy of the calculations, the degree of freedom (df) of relative humidity (RH) was defined as 3; that is, the df of the time-natural cubic spline was 7. Furthermore, dummy variables were set for days of the week and legal holidays to control for the confounding effects of holidays and days of the week (DOW). To determine the relationship between the dampness index and the outpatient visits of dry eye disease (DED) in terms of exposure, lag and response, we constructed the following basic model.

$Y_t \sim Poisson(\mu_t)$

$$Log(Y_t) = \alpha + \beta Relative\ humidity_{t,\ l} + ns(Mean\ temperature, df = 3) + ns(Precipitation, df = 3) + ns(Air\ pressure, df = 3) + ns(WWind\ speed, df = 3) + ns(PM_{2.5}, df = 3) + ns(PM_{10}, df = 3) + ns(SO_2, df = 3) + ns(NO_2, df = 3) + ns(CO, df = 3) + ns(O_3, df = 3) + ns(TTime, df = 7 \times 28) + \gamma DDOWW_t + \delta HHoliday_t$$

In summary, the variable t denotes the number of days during the exposure period, μ_t represents the number of days of DED patients, α represents the intercept, β, γ, and δ represent the coefficients of RH, DOW, and holiday, respectively, and l represents the number of lagged days (28 days). Additionally, DOW is a dummy variable representing several weeks. The term "holiday" refers to Chinese public holidays. The values 0 and 1 represent the nonholiday and holiday periods, respectively. The impact of RH on DED was evaluated via the relative risk (RR) and 95% confidence interval (CI). The single-day lagged effect of RH on specific lagged days was estimated. To illustrate, the RH on the day of the patient's visit was designated lag 0, the RH on the previous day was designated lag 1, and so forth. To ascertain the cumulative-day lag effects, the moving average lag effect of RH was estimated. To illustrate, the RH on lag 0–1 is calculated by lag 0 plus lag 1, and so forth. To assess the applicability of the model and the potential for bias in the autocorrelation of the residuals, residual plots and the partial autocorrelation function (PACF) were employed. The degrees of freedom (df) values were selected on the basis of the minimum sum of the absolute values of the partial autocorrelation function (PACF).

Table S1. Spearman's correlation coefficients of meteorological factors and air pollutants in Shanghai, 2019-2023.

**Variables**	**$PM_{2.5}$**	**PM_{10}**	**SO_2**	**CO**	**NO_2**	**O_3**	**Relative humidity**	**Air pressure**	**Precipitation**	**Wind speed**	**Temperature**
$PM_{2.5}$	1.000*										
PM_{10}	0.792*	1.000*									
SO_2	0.505*	0.627*	1.000*								
CO	0.713*	0.531*	0.411*	1.000*							
NO_2	0.653*	0.586*	0.529*	0.525*	1.000*						
O_3	0.133*	0.169*	0.174*	-0.047†	-0.207*	1.000*					
Relative humidity	-0.126*	-0.467*	-0.433*	-0.037	-0.118*	-0.200*	1.000*				
Air pressure	0.192*	0.298*	0.313*	0.163*	0.381*	-0.280*	-0.469*	1.000*			
Precipitation	-0.238*	-0.498*	-0.469*	-0.052†	-0.246*	-0.171*	0.734*	-0.377*	1.000*		
Wind speed	-0.325*	-0.218*	-0.161*	-0.296*	-0.473*	-0.044*	-0.027	0.019	0.116*	1.000*	
Temperature	-0.312*	-0.303*	-0.275*	-0.293*	-0.450*	0.331*	0.232*	-0.828*	0.183*	-0.060	1.000*

$PM_{2.5}$: particulate matter less than 2.5 μm; PM_{10}: particulate matter less than 10 μm; SO_2: sulphur dioxide; CO: carbon monoxide; NO_2: nitrogen dioxide; O_3: ozone.

*P<0.001.

†P<0.01.

Table S2. Changes in the relative risk of dry eye disease for relative humidity based on the single-day and cumulative-day lag effects model in total in Shanghai, 2019–2023.

Lag days	Single-day lag RR (95%CI)	Lag days	Cumulative-day lag RR (95%CI)
0	1.012 (0.872-1.174)	0-0	1.012 (0.872-1.174)
1	1.011 (0.918-1.114)	0-1	1.023 (0.801-1.306)
2	1.014 (0.953-1.079)	0-2	1.037 (0.767-1.403)
3	1.020 (0.978-1.064)	0-3	1.058 (0.758-1.477)
4	1.028 (0.991-1.066)	0-4	1.088 (0.768-1.541)
5	1.037 (0.999-1.076)	0-5	1.128 (0.792-1.607)
6	1.047 (1.008-1.087)	0-6	1.180 (0.827-1.686)
7	1.056 (1.018-1.096)	0-7	1.247 (0.872-1.783)
8	1.065 (1.028-1.103)	0-8	1.328 (0.925-1.906)
9	1.073 (1.038-1.109)	0-9	1.425 (0.987-2.057)
10	1.079 (1.046-1.114)	0-10	1.538 (1.056-2.238)
11	1.084 (1.050-1.118)	0-11	1.666 (1.132-2.454)
12	1.086 (1.052-1.121)	0-12	1.810 (1.212-2.703)
13	1.087 (1.052-1.123)	0-13	1.967 (1.296-2.986)
14	1.085 (1.049-1.122)	0-14	2.134 (1.380-3.300)
15	1.081 (1.046-1.118)	0-15	2.308 (1.463-3.639)
16	1.076 (1.041-1.111)	0-16	2.482 (1.543-3.993)
17	1.069 (1.035-1.103)	0-17	2.652 (1.617-4.348)
18	1.060 (1.028-1.093)	0-18	2.812 (1.685-4.692)
19	1.051 (1.020-1.084)	0-19	2.956 (1.744-5.011)
20	1.042 (1.010-1.076)	0-20	3.082 (1.794-5.293)
21	1.034 (0.999-1.070)	0-21	3.186 (1.834-5.536)
22	1.027 (0.990-1.065)	0-22	3.271 (1.863-5.742)
23	1.021 (0.983-1.061)	0-23	3.341 (1.884-5.923)
24	1.019 (0.981-1.058)	0-24	3.403 (1.900-6.095)
25	1.020 (0.983-1.058)	0-25	3.471 (1.917-6.283)
26	1.026 (0.986-1.067)	0-26	3.561 (1.943-6.526)
27	1.038 (0.983-1.095)	0-27	3.695 (1.983-6.885)
28	1.057 (0.972-1.150)	0-28	3.907 (2.040-7.485)

Table S3. Changes in the relative risk of dry eye disease for relative humidity based on the single-day and cumulative-day lag effects model in different sex in Shanghai, 2019–2023.

Groups	Lag days	Single-day lag RR (95%CI)	Lag days	Cumulative-day lag RR (95%CI)
Female	0	1.012 (0.868-1.179)	0-0	1.012 (0.868-1.179)
	1	1.009 (0.913-1.115)	0-1	1.021 (0.794-1.313)
	2	1.011 (0.948-1.077)	0-2	1.032 (0.756-1.408)
	3	1.016 (0.972-1.061)	0-3	1.048 (0.743-1.477)
	4	1.023 (0.985-1.062)	0-4	1.072 (0.749-1.535)
	5	1.032 (0.993-1.072)	0-5	1.106 (0.768-1.593)

	6	1.042 (1.002-1.083)	0-6	1.152 (0.798-1.663)
	7	1.052 (1.012-1.093)	0-7	1.212 (0.838-1.752)
	8	1.061 (1.023-1.100)	0-8	1.285 (0.886-1.865)
	9	1.069 (1.034-1.106)	0-9	1.375 (0.942-2.006)
	10	1.076 (1.042-1.112)	0-10	1.479 (1.005-2.178)
	11	1.081 (1.047-1.117)	0-11	1.600 (1.074-2.382)
	12	1.084 (1.049-1.120)	0-12	1.734 (1.148-2.621)
	13	1.085 (1.049-1.122)	0-13	1.882 (1.225-2.892)
	14	1.084 (1.047-1.121)	0-14	2.039 (1.302-3.194)
	15	1.080 (1.044-1.118)	0-15	2.202 (1.378-3.520)
	16	1.074 (1.039-1.111)	0-16	2.366 (1.450-3.859)
	17	1.067 (1.033-1.102)	0-17	2.525 (1.518-4.200)
	18	1.059 (1.026-1.093)	0-18	2.673 (1.578-4.528)
	19	1.050 (1.017-1.083)	0-19	2.805 (1.630-4.829)
	20	1.040 (1.007-1.075)	0-20	2.918 (1.672-5.093)
	21	1.032 (0.996-1.069)	0-21	3.011 (1.705-5.317)
	22	1.024 (0.986-1.063)	0-22	3.083 (1.727-5.503)
	23	1.019 (0.980-1.059)	0-23	3.141 (1.742-5.664)
	24	1.017 (0.978-1.057)	0-24	3.194 (1.753-5.819)
	25	1.018 (0.980-1.058)	0-25	3.252 (1.765-5.992)
	26	1.025 (0.984-1.068)	0-26	3.334 (1.787-6.222)
	27	1.039 (0.983-1.098)	0-27	3.464 (1.825-6.574)
	28	1.061 (0.972-1.157)	0-28	3.674 (1.881-7.175)
Male	0	1.011 (0.862-1.185)	0-0	1.011 (0.862-1.185)
	1	1.016 (0.916-1.127)	0-1	1.027 (0.791-1.334)
	2	1.023 (0.957-1.093)	0-2	1.050 (0.760-1.451)
	3	1.031 (0.985-1.079)	0-3	1.083 (0.758-1.548)
	4	1.040 (1.000-1.082)	0-4	1.127 (0.776-1.636)
	5	1.050 (1.009-1.092)	0-5	1.183 (0.809-1.728)
	6	1.059 (1.017-1.103)	0-6	1.252 (0.855-1.834)
	7	1.068 (1.026-1.111)	0-7	1.337 (0.911-1.962)
	8	1.075 (1.036-1.117)	0-8	1.438 (0.977-2.117)
	9	1.082 (1.044-1.121)	0-9	1.556 (1.050-2.305)
	10	1.087 (1.051-1.124)	0-10	1.691 (1.131-2.528)
	11	1.090 (1.054-1.127)	0-11	1.843 (1.217-2.790)
	12	1.091 (1.055-1.129)	0-12	2.011 (1.309-3.091)
	13	1.091 (1.053-1.130)	0-13	2.194 (1.402-3.432)
	14	1.088 (1.050-1.128)	0-14	2.388 (1.496-3.810)
	15	1.084 (1.046-1.124)	0-15	2.589 (1.589-4.219)
	16	1.079 (1.042-1.117)	0-16	2.793 (1.678-4.649)
	17	1.072 (1.037-1.109)	0-17	2.994 (1.762-5.087)
	18	1.064 (1.030-1.100)	0-18	3.187 (1.841-5.517)
	19	1.056 (1.022-1.091)	0-19	3.365 (1.912-5.923)
	20	1.048 (1.013-1.084)	0-20	3.526 (1.974-6.295)

21	1.040 (1.003-1.078)	0-21	3.666 (2.028-6.627)
22	1.033 (0.993-1.074)	0-22	3.786 (2.071-6.920)
23	1.027 (0.987-1.070)	0-23	3.889 (2.106-7.184)
24	1.024 (0.984-1.066)	0-24	3.983 (2.133-7.437)
25	1.024 (0.984-1.065)	0-25	4.078 (2.159-7.703)
26	1.027 (0.985-1.072)	0-26	4.189 (2.189-8.017)
27	1.035 (0.977-1.097)	0-27	4.337 (2.227-8.447)
28	1.049 (0.959-1.148)	0-28	4.549 (2.267-9.129)

Table S4. Changes in the relative risk of dry eye disease for relative humidity based on the single-day and cumulative-day lag effects model in different ages in Shanghai, 2019–2023.

**Groups**	**Lag days**	**Single-day lag RR (95%CI)**	**Lag days**	**Cumulative-day lag RR (95%CI)**
0-18 years	0	1.077 (0.777-1.492)	0-0	1.077 (0.777-1.492)
	1	1.039 (0.841-1.285)	0-1	1.119 (0.655-1.913)
	2	1.016 (0.888-1.163)	0-2	1.137 (0.587-2.205)
	3	1.004 (0.914-1.102)	0-3	1.142 (0.550-2.371)
	4	1.000 (0.921-1.084)	0-4	1.141 (0.532-2.448)
	5	1.001 (0.923-1.087)	0-5	1.143 (0.526-2.483)
	6	1.008 (0.927-1.095)	0-6	1.152 (0.528-2.513)
	7	1.017 (0.937-1.103)	0-7	1.171 (0.535-2.563)
	8	1.027 (0.951-1.109)	0-8	1.202 (0.545-2.649)
	9	1.037 (0.965-1.114)	0-9	1.246 (0.559-2.779)
	10	1.046 (0.977-1.120)	0-10	1.304 (0.575-2.958)
	11	1.054 (0.984-1.127)	0-11	1.374 (0.591-3.190)
	12	1.058 (0.988-1.134)	0-12	1.454 (0.608-3.477)
	13	1.060 (0.987-1.138)	0-13	1.541 (0.622-3.815)
	14	1.058 (0.984-1.138)	0-14	1.631 (0.633-4.198)
	15	1.053 (0.980-1.133)	0-15	1.718 (0.640-4.611)
	16	1.045 (0.974-1.122)	0-16	1.796 (0.641-5.029)
	17	1.035 (0.966-1.108)	0-17	1.858 (0.637-5.422)
	18	1.022 (0.956-1.092)	0-18	1.899 (0.626-5.755)
	19	1.008 (0.943-1.078)	0-19	1.914 (0.611-6.000)
	20	0.994 (0.927-1.066)	0-20	1.903 (0.590-6.141)
	21	0.982 (0.910-1.058)	0-21	1.868 (0.565-6.182)
	22	0.971 (0.896-1.053)	0-22	1.814 (0.536-6.144)
	23	0.965 (0.887-1.049)	0-23	1.750 (0.505-6.060)
	24	0.964 (0.886-1.048)	0-24	1.686 (0.476-5.976)
	25	0.970 (0.893-1.053)	0-25	1.635 (0.450-5.943)
	26	0.986 (0.902-1.077)	0-26	1.612 (0.431-6.027)
	27	1.014 (0.899-1.144)	0-27	1.636 (0.422-6.345)
	28	1.059 (0.878-1.278)	0-28	1.733 (0.420-7.154)
19-60 years	0	1.006 (0.863-1.173)	0-0	1.006 (0.863-1.173)
	1	1.004 (0.908-1.109)	0-1	1.010 (0.785-1.300)

	2	1.005 (0.943-1.071)	0-2	1.016 (0.744-1.387)
	3	1.010 (0.967-1.056)	0-3	1.026 (0.727-1.448)
	4	1.018 (0.980-1.057)	0-4	1.044 (0.729-1.496)
	5	1.027 (0.988-1.067)	0-5	1.072 (0.744-1.545)
	6	1.037 (0.997-1.078)	0-6	1.112 (0.770-1.605)
	7	1.047 (1.008-1.087)	0-7	1.164 (0.805-1.682)
	8	1.056 (1.019-1.095)	0-8	1.229 (0.847-1.783)
	9	1.065 (1.029-1.102)	0-9	1.309 (0.897-1.909)
	10	1.072 (1.038-1.107)	0-10	1.403 (0.954-2.065)
	11	1.078 (1.044-1.113)	0-11	1.512 (1.016-2.250)
	12	1.081 (1.046-1.117)	0-12	1.635 (1.083-2.468)
	13	1.082 (1.047-1.119)	0-13	1.769 (1.152-2.717)
	14	1.082 (1.045-1.119)	0-14	1.914 (1.223-2.994)
	15	1.079 (1.042-1.116)	0-15	2.064 (1.293-3.295)
	16	1.074 (1.038-1.110)	0-16	2.216 (1.360-3.610)
	17	1.067 (1.033-1.102)	0-17	2.364 (1.423-3.929)
	18	1.059 (1.027-1.093)	0-18	2.505 (1.480-4.238)
	19	1.051 (1.018-1.084)	0-19	2.632 (1.531-4.524)
	20	1.042 (1.008-1.077)	0-20	2.742 (1.573-4.779)
	21	1.034 (0.998-1.070)	0-21	2.834 (1.607-4.998)
	22	1.027 (0.989-1.066)	0-22	2.909 (1.632-5.185)
	23	1.021 (0.982-1.062)	0-23	2.971 (1.651-5.349)
	24	1.019 (0.980-1.059)	0-24	3.028 (1.665-5.507)
	25	1.020 (0.983-1.060)	0-25	3.090 (1.680-5.682)
	26	1.027 (0.986-1.070)	0-26	3.172 (1.703-5.909)
	27	1.039 (0.983-1.099)	0-27	3.298 (1.741-6.247)
	28	1.060 (0.972-1.156)	0-28	3.495 (1.792-6.816)
>60 years	0	1.017 (0.858-1.205)	0-0	1.017 (0.858-1.205)
	1	1.023 (0.915-1.142)	0-1	1.040 (0.786-1.375)
	2	1.030 (0.960-1.106)	0-2	1.071 (0.759-1.513)
	3	1.040 (0.990-1.092)	0-3	1.114 (0.761-1.631)
	4	1.050 (1.006-1.095)	0-4	1.169 (0.785-1.741)
	5	1.060 (1.015-1.106)	0-5	1.239 (0.826-1.859)
	6	1.070 (1.024-1.118)	0-6	1.326 (0.881-1.994)
	7	1.079 (1.034-1.126)	0-7	1.430 (0.949-2.157)
	8	1.087 (1.043-1.132)	0-8	1.554 (1.026-2.354)
	9	1.093 (1.052-1.136)	0-9	1.699 (1.114-2.592)
	10	1.098 (1.058-1.139)	0-10	1.866 (1.211-2.875)
	11	1.100 (1.061-1.141)	0-11	2.053 (1.314-3.207)
	12	1.101 (1.061-1.142)	0-12	2.261 (1.423-3.590)
	13	1.099 (1.059-1.142)	0-13	2.485 (1.535-4.024)
	14	1.096 (1.055-1.139)	0-14	2.724 (1.646-4.505)
	15	1.090 (1.049-1.133)	0-15	2.970 (1.755-5.026)
	16	1.083 (1.044-1.125)	0-16	3.217 (1.858-5.571)

17	1.075 (1.037-1.115)	0-17	3.459 (1.954-6.122)
18	1.066 (1.029-1.104)	0-18	3.687 (2.041-6.659)
19	1.056 (1.019-1.094)	0-19	3.894 (2.117-7.160)
20	1.047 (1.009-1.086)	0-20	4.075 (2.182-7.611)
21	1.038 (0.997-1.079)	0-21	4.228 (2.234-8.003)
22	1.030 (0.987-1.074)	0-22	4.354 (2.273-8.341)
23	1.024 (0.980-1.070)	0-23	4.458 (2.301-8.641)
24	1.021 (0.977-1.066)	0-24	4.551 (2.321-8.925)
25	1.021 (0.979-1.065)	0-25	4.648 (2.341-9.228)
26	1.026 (0.980-1.074)	0-26	4.768 (2.368-9.601)
27	1.036 (0.974-1.102)	0-27	4.940 (2.408-10.134)
28	1.053 (0.956-1.159)	0-28	5.201 (2.457-11.007)

Table S5. Changes in the relative risk of DED for humidity based on the single-day and cumulative-day lag effects model in warm and cold season in Shanghai, 2019–2023.

Groups	Lag days	Single-day lag RR (95%CI)	Lag days	Cumulative-day lag RR (95%CI)
Warm season	0	1.146 (0.898-1.461)	0-0	1.146 (0.898-1.461)
	1	1.108 (0.941-1.304)	0-1	1.270 (0.848-1.902)
	2	1.085 (0.971-1.214)	0-2	1.378 (0.830-2.287)
	3	1.074 (0.983-1.173)	0-3	1.480 (0.836-2.620)
	4	1.071 (0.984-1.164)	0-4	1.584 (0.857-2.930)
	5	1.074 (0.984-1.171)	0-5	1.701 (0.888-3.259)
	6	1.081 (0.987-1.183)	0-6	1.838 (0.926-3.650)
	7	1.090 (0.994-1.196)	0-7	2.004 (0.968-4.146)
	8	1.100 (1.002-1.207)	0-8	2.204 (1.015-4.787)
	9	1.110 (1.011-1.218)	0-9	2.446 (1.065-5.616)
	10	1.117 (1.017-1.228)	0-10	2.732 (1.117-6.681)
	11	1.122 (1.019-1.235)	0-11	3.066 (1.171-8.031)
	12	1.124 (1.019-1.239)	0-12	3.446 (1.222-9.712)
	13	1.121 (1.015-1.239)	0-13	3.864 (1.269-11.765)
	14	1.115 (1.009-1.233)	0-14	4.309 (1.307-14.205)
	15	1.105 (1.000-1.221)	0-15	4.763 (1.333-17.011)
	16	1.092 (0.989-1.205)	0-16	5.200 (1.345-20.111)
	17	1.076 (0.977-1.185)	0-17	5.595 (1.339-23.377)
	18	1.058 (0.963-1.162)	0-18	5.920 (1.316-26.636)
	19	1.040 (0.948-1.140)	0-19	6.155 (1.276-29.701)
	20	1.022 (0.933-1.120)	0-20	6.291 (1.221-32.411)
	21	1.006 (0.918-1.103)	0-21	6.332 (1.156-34.683)
	22	0.994 (0.907-1.091)	0-22	6.296 (1.085-36.541)
	23	0.988 (0.900-1.083)	0-23	6.218 (1.014-38.140)
	24	0.988 (0.902-1.083)	0-24	6.144 (0.949-39.768)
	25	0.998 (0.913-1.092)	0-25	6.134 (0.899-41.870)

	26	1.021 (0.931-1.120)	0-26	6.261 (0.869-45.128)
	27	1.060 (0.949-1.182)	0-27	6.634 (0.868-50.704)
	28	1.120 (0.962-1.303)	0-28	7.428 (0.906-60.900)
Cold season	0	0.971 (0.796-1.185)	0-0	0.971 (0.796-1.185)
	1	0.978 (0.857-1.115)	0-1	0.950 (0.683-1.320)
	2	0.987 (0.906-1.076)	0-2	0.938 (0.623-1.412)
	3	0.999 (0.938-1.064)	0-3	0.937 (0.593-1.480)
	4	1.012 (0.957-1.071)	0-4	0.949 (0.585-1.539)
	5	1.027 (0.970-1.087)	0-5	0.974 (0.590-1.608)
	6	1.042 (0.983-1.104)	0-6	1.015 (0.606-1.700)
	7	1.057 (0.997-1.120)	0-7	1.072 (0.630-1.826)
	8	1.071 (1.012-1.133)	0-8	1.148 (0.661-1.995)
	9	1.084 (1.027-1.145)	0-9	1.245 (0.699-2.217)
	10	1.096 (1.040-1.156)	0-10	1.364 (0.744-2.502)
	11	1.106 (1.050-1.166)	0-11	1.510 (0.796-2.862)
	12	1.115 (1.057-1.175)	0-12	1.683 (0.856-3.309)
	13	1.121 (1.063-1.183)	0-13	1.887 (0.923-3.856)
	14	1.125 (1.066-1.187)	0-14	2.123 (0.997-4.519)
	15	1.127 (1.068-1.188)	0-15	2.392 (1.078-5.306)
	16	1.126 (1.070-1.186)	0-16	2.694 (1.166-6.224)
	17	1.124 (1.069-1.181)	0-17	3.027 (1.260-7.272)
	18	1.119 (1.067-1.173)	0-18	3.387 (1.359-8.441)
	19	1.113 (1.063-1.166)	0-19	3.771 (1.464-9.714)
	20	1.106 (1.056-1.158)	0-20	4.170 (1.571-11.068)
	21	1.098 (1.047-1.152)	0-21	4.578 (1.680-12.481)
	22	1.090 (1.037-1.145)	0-22	4.989 (1.786-13.936)
	23	1.081 (1.028-1.138)	0-23	5.395 (1.887-15.422)
	24	1.074 (1.021-1.130)	0-24	5.795 (1.982-16.946)
	25	1.068 (1.017-1.122)	0-25	6.191 (2.068-18.531)
	26	1.065 (1.010-1.123)	0-26	6.592 (2.147-20.238)
	27	1.064 (0.991-1.142)	0-27	7.013 (2.215-22.204)
	28	1.067 (0.956-1.190)	0-28	7.483 (2.263-24.747)

Table S6. Extremely low and high relative humidity on the cumulative relative risk of dry eye disease over different lag days stratified by patient's sex, age and season

Groups	Extremely low relative humidity (5th)					Extremely high relative humidity (95th)				
	Lag 0-1	Lag 0-7	Lag 0-14	Lag 0-21	Lag 0-28	Lag 0-1	Lag 0-7	Lag 0-14	Lag 0-21	Lag 0-28
Total	1.017 (0.848-1.220)	1.178 (0.903-1.538)	1.757 (1.271-2.430)	2.367 (1.570-3.570)	2.755 (1.699-4.467)	1.027 (0.770-1.370)	1.297 (0.850-1.978)	2.445 (1.462-4.089)	3.923 (2.045-7.526)	4.990 (2.318-10.742)
Sex										
Female	1.016 (0.842-1.225)	1.153 (0.877-1.517)	1.699 (1.217-2.372)	2.269 (1.487-3.464)	2.632 (1.600-4.329)	1.025 (0.762-1.379)	1.126 (0.733-1.732)	2.317 (1.365-3.934)	3.669 (1.876-7.176)	4.640 (2.107-10.219)
Male	1.020 (0.840-1.239)	1.241 (0.933-1.650)	1.910 (1.349-2.704)	2.627 (1.692-4.080)	3.085 (1.838-5.178)	1.032 (0.758-1.405)	1.409 (0.896-2.214)	2.791 (1.609-4.843)	4.628 (2.302-9.305)	5.971 (2.626-13.576)
Age（in years）										
0-18	1.087 (0.730-1.620)	1.124 (0.628-2.013)	1.439 (0.712-2.906)	1.592 (0.654-3.875)	1.505 (0.524-4.320)	1.142 (0.607-2.149)	1.204 (0.478-3.035)	1.780 (0.584-5.431)	2.090 (0.509-8.573)	1.912 (0.359-10.185)
19-60	1.007 (0.835-1.215)	1.119 (0.851-1.472)	1.620 (1.161-2.260)	2.170 (1.423-3.308)	2.536 (1.543-4.167)	1.012 (0.751-1.363)	1.196 (0.774-1.847)	2.150 (1.268-3.646)	3.417 (1.750-6.672)	4.375 (1.990-9.619)
>60	1.029 (0.836-1.267)	1.305 (0.961-1.771)	2.106 (1.449-3.063)	2.921 (1.818-4.695)	3.408 (1.951-5.951)	1.047 (0.753-1.455)	1.525 (0.940-2.476)	3.260 (1.800-5.903)	5.477 (2.58-11.624)	6.991 (2.887-16.930)
Season										
Warm season	1.200 (0.882-1.633)	1.699 (0.976-2.957)	3.046 (1.227-7.564)	4.085 (1.117-14.94)	4.613 (0.927-22.949)	1.316 (0.827-2.094)	2.224 (0.964-5.131)	5.365 (1.361-21.15)	8.351 (1.181-59.039)	10.034 (0.893-112.801)
Cold season	0.962 (0.754-1.229)	1.053 (0.709-1.564)	1.750 (0.998-3.068)	3.098 (1.470-6.528)	4.463 (1.835-10.857)	0.939 (0.626-1.406)	1.089 (0.566-2.097)	2.523 (0.996-6.390)	6.494 (1.892-22.290)	11.882 (2.730-51.720)

Figure S1. Daily changes in DED outpatient visits, meteorological factors, and air pollutants in Shanghai from 2019–2023.

A

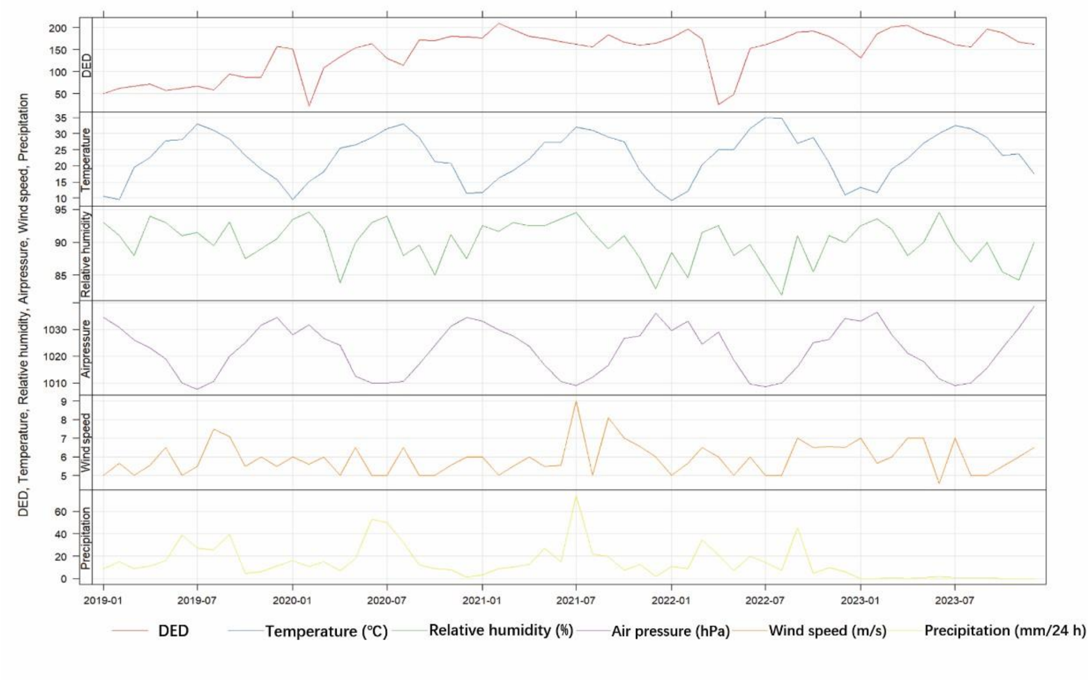

B

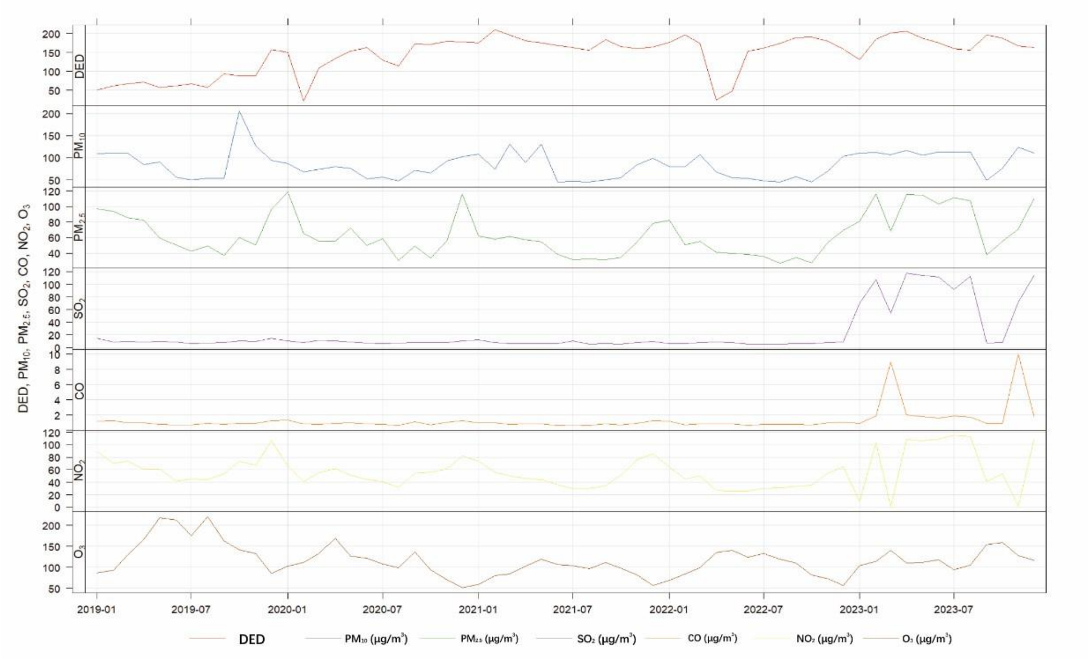

Figure S2. Violin and boxplots showing the variations in meteorological factors, air pollutants, and DED outpatient visits in the warm season and cold season from 2019–2023. Distribution mode of the five meteorological factors between the warm season and cold season (A). The

distribution mode of the six air pollutants between the warm season and cold season (B). The distribution of DED outpatient visits between the warm season and cold season (C).

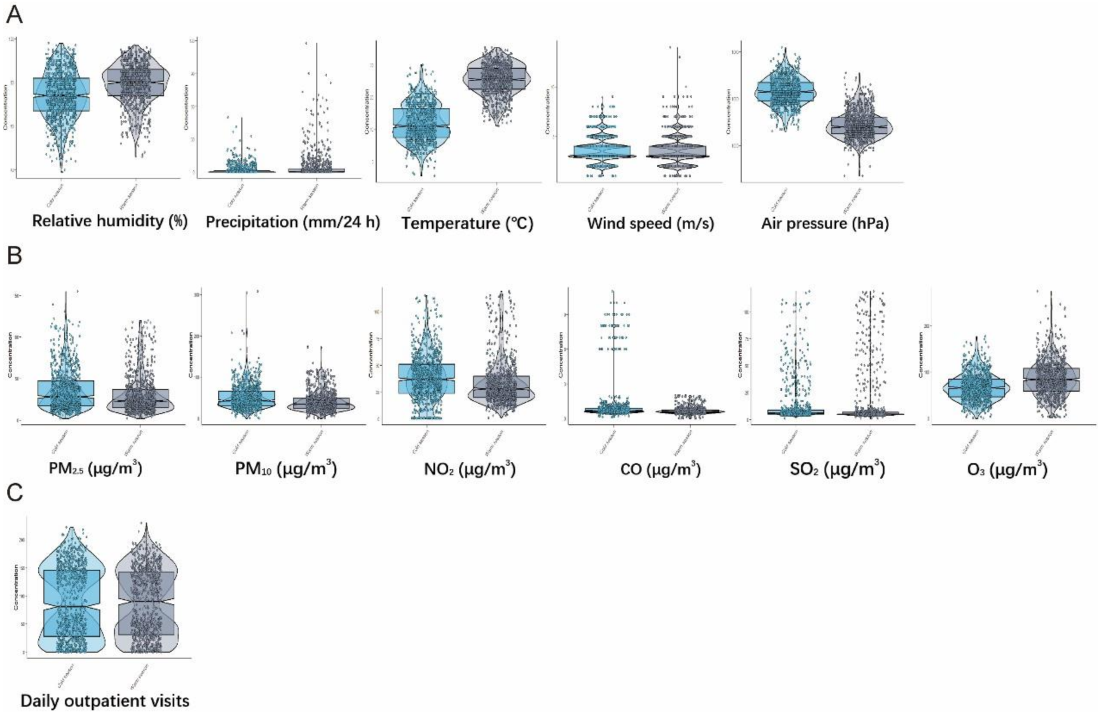

Figure S3. The sample size testing to examine the relationship between RH and DED outpatient visits.

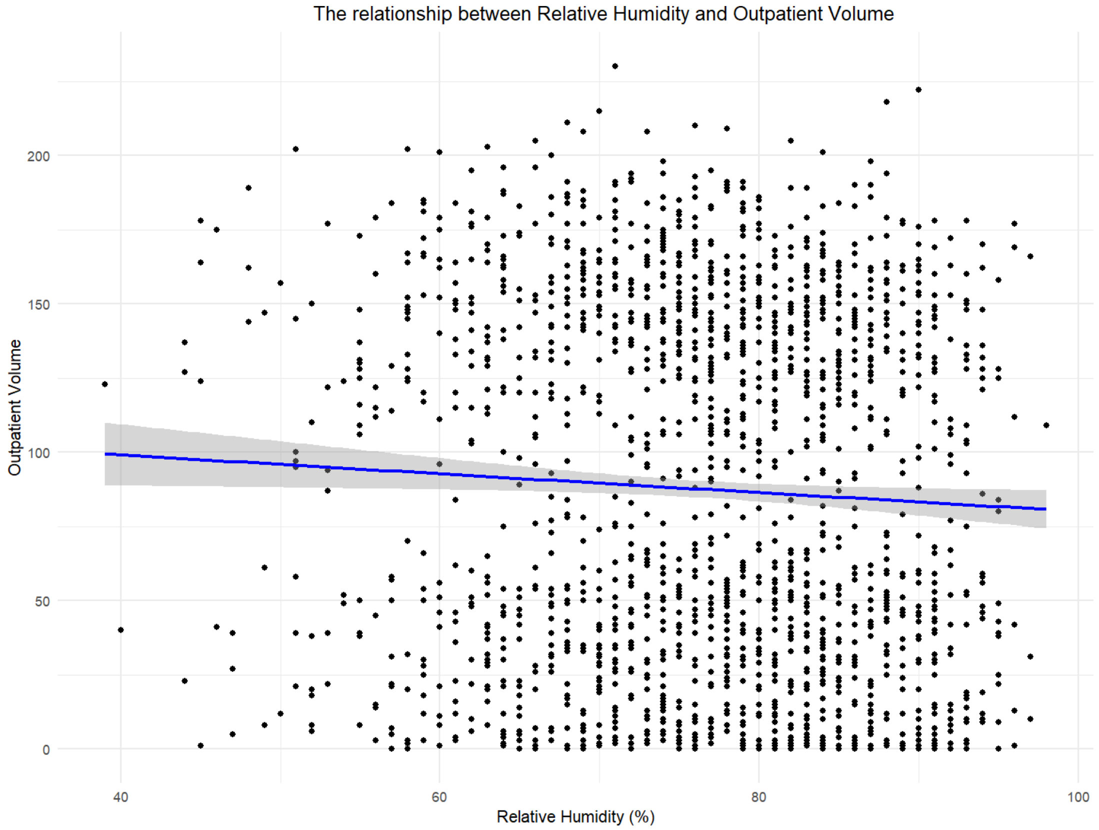

Figure S4. The effect of RH on the relative risk of DED during outpatient visits. 3D illustration showing the correlation between RH and daily admissions of DED patients at various RH levels and lag days (A). Contour plot showing the exposure–lag–response relationship between RH and DED outpatient visits on various lag days (B). The blue areas indicate relative risks (RR)<1; the red areas indicate relative risks (RR)>1.

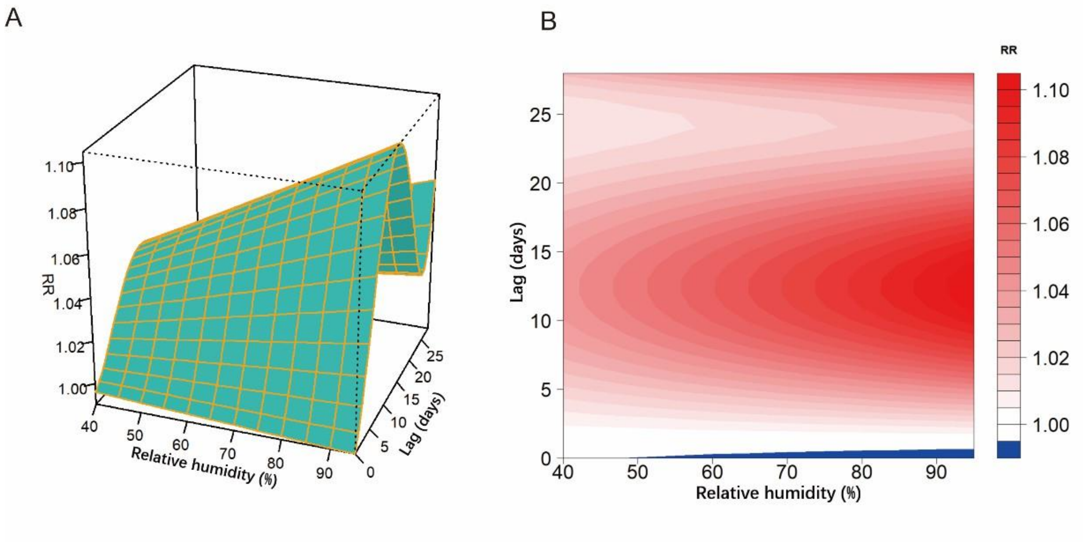

Figure S5. The effect of RH on the relative risk of DED during outpatient visits. The single-day lag pattern (A). The cumulative-day lag effect pattern. The gray areas indicate the 95% confidence intervals (CIs) (B).

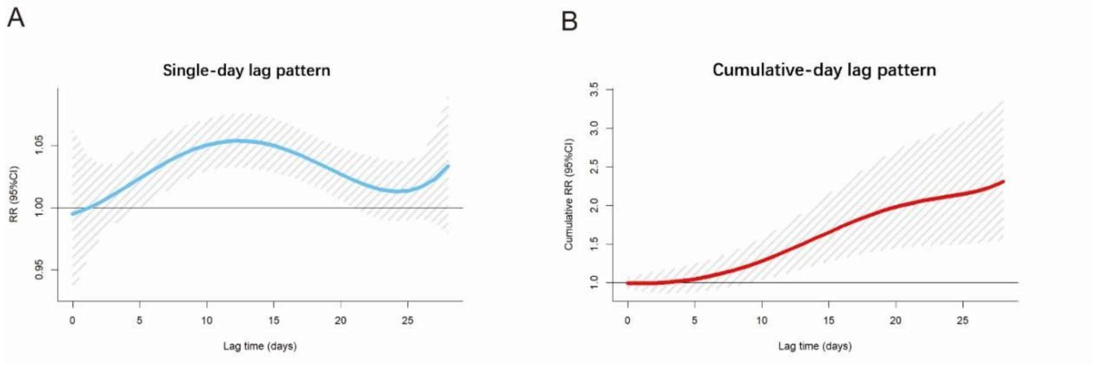

Figure S6. The sensitivity analysis between the various value of df (6-8) for date time and df (3-5) for air pressure, wind speed, mean temperature, precipitation, and relative humidity on the effects of relative humidity on dry eye disease outpatient visits at the single-day lag pattern and cumulative-day lag effect pattern.

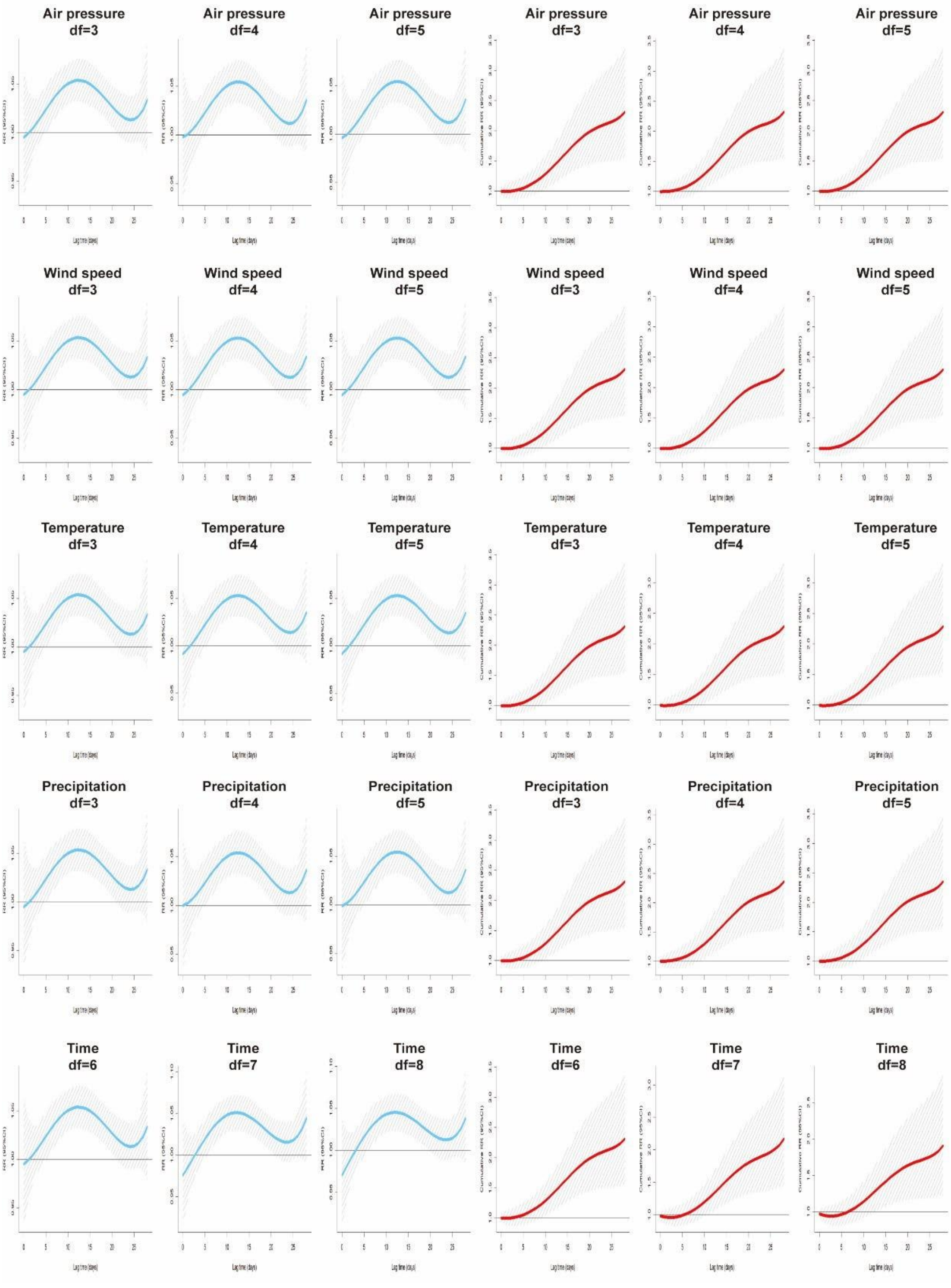

Figure S7. The sensitivity analysis between the various value of df (3-5) for $PM_{2.5}$, PM_{10}, O_3, NO_2, CO, and SO_2 on effects of relative humidity on the dry eye disease outpatient visits at the single-day lag pattern and cumulative-day lag effect pattern.

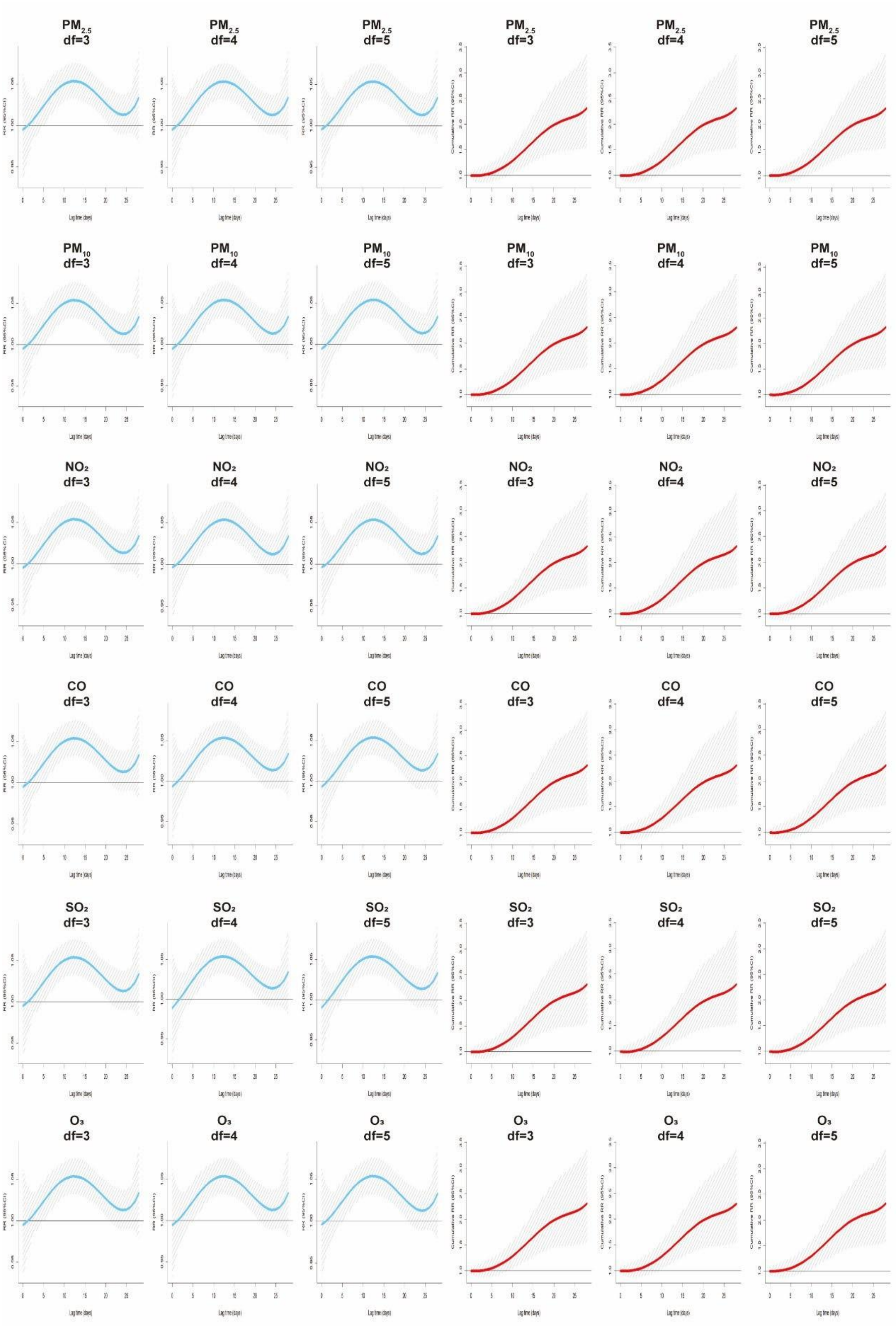

Figure S8. The sensitivity analysis between the changing maximum lag days (28-31) on effects of relative humidity on the dry eye disease outpatient visits at the single-day lag pattern and cumulative-day lag effect pattern.

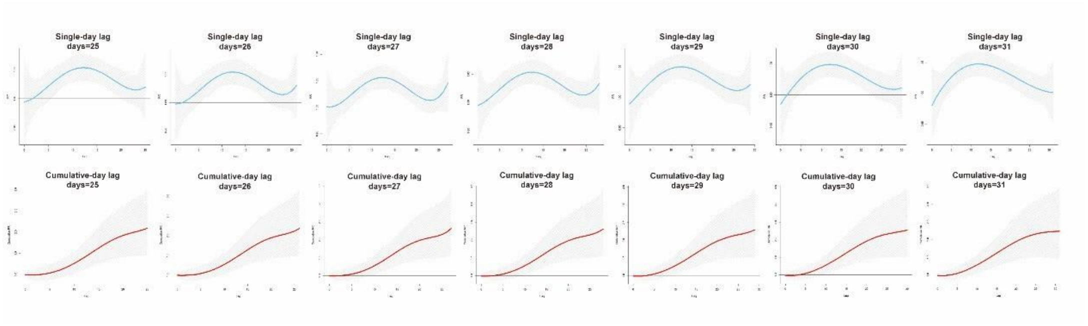

Supplement: Online Supplementary Document [file jogh-15-04142-s001.pdf]
